# Diet of a threatened rattlesnake (eastern massasauga) revealed by DNA metabarcoding

**DOI:** 10.1002/ece3.10029

**Published:** 2023-04-26

**Authors:** Alyssa Swinehart, Charlyn Partridge, Amy Russell, Arin Thacker, Jennifer Kovach, Jennifer Moore

**Affiliations:** ^1^ Biology Department Grand Valley State University Allendale Michigan USA; ^2^ Annis Water Resources Institute Grand Valley State University Muskegon Michigan USA

**Keywords:** diet analysis, DNA metabarcoding, rattlesnake, reptiles, *Sistrurus catenatus*, threatened species

## Abstract

Characterizing the diet of imperiled species using minimally invasive methods is crucial to understanding their ecology and conservation requirements. Here, we apply a DNA metabarcoding approach to study the diet of the eastern massasauga rattlesnake (*Sistrurus catenatus*), a Federally Threatened snake found throughout the Great Lakes region. Eighty‐three fecal samples collected across 10 different massasauga populations located in Michigan, USA, were sequenced, with 70 samples containing prey DNA. We used universal metazoan primers and developed a host‐specific oligonucleotide blocker to characterize their diet. We identified at least 12 different prey species, with eastern massasaugas exhibiting opportunistic feeding and a strong preference towards small mammals. Meadow voles (*Microtus pennsylvanicus*) were the most common prey item (70% of diet) followed by the northern short‐tailed shrew (*Blarina brevicauda*) and masked shrew (*Sorex cinereus*; 15.7% of diet each), along with occasional bird and snake prey. Adult individuals exhibited a more generalized diet, consuming a larger number of prey taxa on average. Younger snakes consumed a smaller variety of prey items and tended to consume smaller‐sized mammals such as masked shrews (*Sorex cinereus*) and northern short‐tailed shrews (*Blarina brevicauda*). We conclude that small mammals are a crucial part of eastern massasauga rattlesnake diet and recommend this be taken into consideration when conservation strategies are developed. The methods developed in this study can be applied to other reptile species, providing an accurate, minimally invasive, and thorough diet assessment for at‐risk reptile species.

## Graphical abstract

We applied a DNA metabarcoding approach to study the diet of the threatened eastern massasauga rattlesnake (*Sistrurus catenatus*). We identified at least 12 different prey species, with eastern massasaugas exhibiting opportunistic feeding and a strong preference towards small mammals. The methods developed in this study can be applied to other reptile species, providing an accurate, minimally invasive, and thorough diet assessment for at‐risk species.

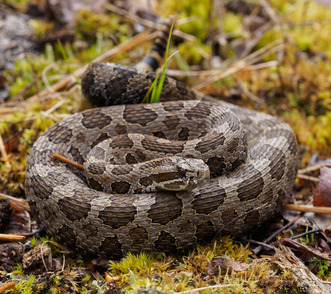

## 1 INTRODUCTION

Molecular characterizations of the diets of imperiled species are becoming increasingly common in ecological studies, allowing crucial food sources or feeding preferences of declining populations to be identified. Obtaining dietary information can indicate the current state and health of the ecosystem and identify if a predator is a specialist or generalist consumer. Compared to generalist consumers, predators with specialist or limited diets are more vulnerable to declines due to limited numbers of suitable prey. If a threatened predator's diet is limited, identifying its preferred food source can help guide the conservation of declining populations (Pompanon et al., 2012). Reptiles are facing global declines (Böhm et al., 2013; Gibbons et al., 2000; Zipkin et al., 2020), with dietary information for at‐risk species often lacking as a consequence. Characterizing diet becomes especially challenging for predatory reptiles, such as snakes, whose feeding events are cryptic and infrequent.

Snake diets have primarily been assessed by examination of stomach contents from wild individuals and museum specimens, or of feces for identifiable remains of prey (hair, scales, skulls, etc.). While these techniques can provide a starting point for diet characterization, they require specialized taxonomic expertise and have the potential for severe biases (Glaudas et al., 2017; Symondson, 2002). For example, many reptiles consume prey that are soft‐bodied or easily digestible (e.g., invertebrates; Brown et al., 2012); detection of these prey items would be impossible by relying solely on morphological identification of remains. Additionally, traditional methods requiring euthanasia for examination of stomach or gut contents are not an option for at‐risk wild individuals due to ethical considerations. An alternative, minimally invasive approach to assessing diet is through analysis of feces using DNA metabarcoding (King et al., 2008; Valentini et al., 2009).

Increased accessibility to high‐throughput sequencing technology, expansion of reference sequences in public databases, and the development of universal primers have drastically improved the success of DNA metabarcoding studies of vertebrate diets (Porter & Hajibabaei, 2018). Obtaining dietary information from fecal samples containing highly degraded prey DNA is now feasible as these primers target short DNA regions. The most commonly used barcode marker for targeting metazoan taxa is the mitochondrial cytochrome c oxidase subunit 1 gene (CO1) (Hebert et al., 2003). The CO1 region has faced criticism for not being truly universal due to potential taxonomic biases (Rubbmark et al., 2018), yet is still accepted as the most suitable barcode region for general metazoan metabarcoding down to the species level (Andújar et al., 2018; de Sousa et al., 2019). Despite the promises of DNA metabarcoding‐based diet analyses, these methods have yet to be commonly applied outside of mammalian systems or arthropod specialist consumers (Bohmann et al., 2018; Deagle et al., 2009; Kartzinel & Pringle, 2015; Shehzad et al., 2012). Reptiles are heavily underrepresented in DNA metabarcoding studies, and only a few have implemented these approaches to study the diet of several lizards (Brown et al., 2012; Kartzinel & Pringle, 2015; Pereira et al., 2019) or snake species (Falk & Reed, 2015) with varying success in amplifying the CO1 region.

The eastern massasauga rattlesnake (*Sistrurus catenatus*) is a wetland species that occurs throughout the Great Lakes region (Seigel, 1986). Massasauga populations have declined across their range, and the species is listed as Federally Threatened under the U.S. Endangered Species Act (U.S. Fish and Wildlife Service, 2016) and the Canadian Species at Risk Act (Committee on the Status of Endangered Wildlife in Canada, 2002). Habitat loss and fragmentation, vegetative succession, road mortalities, and human conflict have contributed to population declines (U.S. Fish and Wildlife Service, 2016). Following drastic declines throughout its range, the majority of the remaining viable populations are located in Michigan, USA.

**FIGURE 1:**
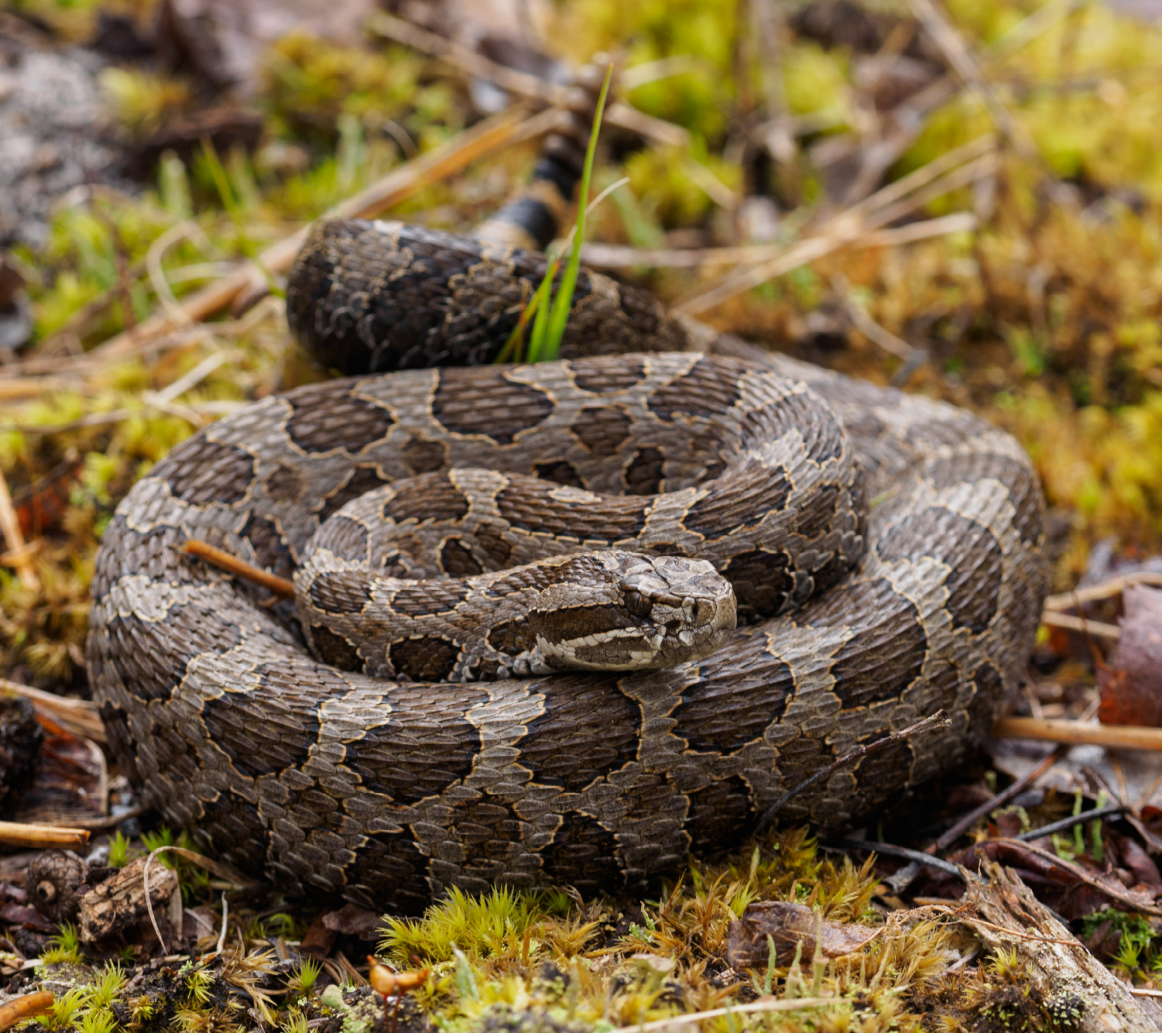
Adult eastern massasauga rattlesnake basking in a wetland (photo credit: Eric McCluskey).

Characteristic of many reptiles, eastern massasaugas possess cryptic coloration and behavior, which, in combination with their threatened status, make them rare and difficult to detect (Figure 1). Thus, feeding observations of this species in the wild are rare, and identification of eastern massasauga diet composition has been limited to opportunistic regurgitations, analyses of fecal and gut contents, and feeding trials (Holycross & Mackessy, 2002; Keenlyne & Beer, 1973; Shepard et al., 2004; Weatherhead et al., 2009). Across their range, these analyses have revealed that the majority of eastern massasauga diet consists of small mammals such as voles, shrews, and mice while occasionally including birds and other snakes (Keenlyne & Beer, 1973; Shepard et al., 2004; Weatherhead et al., 2009; Table 1). Dietary data are currently lacking for Michigan populations, with only two known studies focusing on museum specimens (Hallock, 1991) and opportunistic regurgitations from wild individuals (Tetzlaff et al., 2015). As gape‐limited predators, viperid snakes can exhibit ontogenetic shifts in diet (Glaudas et al., 2008). Juvenile viperids may feed on smaller ectothermic prey such as other snakes and invertebrates, later switching to a more mammal‐dominated diet (Glaudas et al., 2008). However, there is conflicting evidence on the possibility of ontogenetic dietary shifts for massasaugas. In some parts of their range, only neonate and juvenile massasaugas have been documented to consume other snake species, switching primarily to mammals as adults (Keenlyne & Beer, 1973); however, dietary shifts were not evident in Ontario and Ohio massasauga populations (Weatherhead et al., 2009). In feeding trials conducted with neonate eastern massasaugas, snakes preferred other neonate snake prey, but regurgitations from captured free‐ranging individuals only consisted of shrews and voles (Shepard et al., 2004).

**TABLE 1:** List of previously recorded prey items for the eastern massasauga rattlesnake from previous studies using opportunistic regurgitations, gut content analysis, and fecal dissections.

Class	Consumed prey	Common name	Location	Source
Amphibia	Unidentified anuran spp.		Michigan	Hallock (1991), Ruthven (1911)
Aves	*Toxostoma rufum*	Brown thrasher	Michigan	Tetzlaff et al. (2015)
Aves	Unidentified spp.		Michigan	Hallock (1991)
Aves	*Agelaius phoeniceus*	Red‐winged blackbird	Wisconsin	Keenlyne and Beer (1973)
Insecta	Unidentified spp.		Michigan	Hallock (1991)
Mammalia	*Microtus ochrogaster*	Prairie vole	Illinois	Shepard et al. (2004)
Mammalia	*Blarina carolinensis*	Southern short‐tailed shrew	Illinois	Shepard et al. (2004)
Mammalia	Unidentified murid spp.		Michigan	Tetzlaff et al. (2015)
Mammalia	*Microtus* spp.		Michigan	Hallock (1991)
Mammalia	*Napaeozapus insignis*	Woodland jumping mouse	Michigan	Hallock (1991)
Mammalia	*Peromyscus* spp.		Michigan	Hallock (1991)
Mammalia	*Peromyscus maniculatus*	Deer mouse	Ohio	Weatherhead et al. (2009)
Mammalia	*Tamiasciurus hudsonicus*	American red squirrel	Michigan, Ontario	Tetzlaff et al. (2015), Weatherhead et al. (2009)
Mammalia	*Myodes gapperi*	Southern red‐backed vole	Michigan, Ontario	Hallock (1991), Weatherhead et al. (2009)
Mammalia	*Blarina brevicauda*	Northern short‐tailed shrew	Michigan, Ontario, Ohio	Hallock (1991), Weatherhead et al. (2009)
Mammalia	*Microtus pennsylvanicus*	Meadow vole	Michigan, Ontario, Ohio, Wisconsin	Hallock (1991), Weatherhead et al. (2009), Keenlyne and Beer (1973)
Mammalia	*Sylvilagus floridanus*	Eastern cottontail	Ohio	Weatherhead et al. (2009)
Mammalia	*Sciurus niger*	Eastern fox squirrel	Ohio	Weatherhead et al. (2009)
Mammalia	*Glaucomys sabrinus*	Northern flying squirrel	Ontario	Weatherhead et al. (2009)
Mammalia	*Lepus americanus*	Snowshoe hare	Ontario	Weatherhead et al. (2009)
Mammalia	*Tamias striatus*	Eastern chipmunk	Ontario, Ohio	Weatherhead et al. (2009)
Mammalia	*Sorex cinereus*	Masked shrew	Ontario, Ohio, Wisconsin	Weatherhead et al. (2009), Keenlyne and Beer (1973)
Mammalia	*Zapus hudsonius*	Meadow jumping mouse	Ontario, Ohio, Wisconsin	Weatherhead et al. (2009), Keenlyne and Beer (1973)
Mammalia	*Peromyscus leucopus*	White‐footed mouse	Wisconsin	Keenlyne and Beer (1973)
Reptilia	*Storeria dekayi*	Brown snake	Michigan	Hallock (1991)
Reptilia	*Sistrurus catenatus*	Eastern massasauga	Michigan	Ruthven (1911), Hallock (1991)
Reptilia	*Storeria o. occipitomaculata*	Northern red‐bellied snake	Michigan	Tetzlaff et al. (2015)
Reptilia	Unidentified snake spp.		Michigan, Ontario, Wisconsin	Hallock (1991), Ruthven (1911), Weatherhead et al. (2009), Keenlyne and Beer (1973)
Reptilia	*Thamnophis* spp.	Garter snake	Michigan, Wisconsin	Hallock (1991), Keenlyne and Beer (1973), Tetzlaff et al. (2015)

*Note*: Records listed under the consumed prey column are the most specific identification that was possible.

Snake species that rely on venom for prey capture, such as ambush predators, may have a more specialized diet based on their prey‐specific venom, while others with less complex venoms may follow a generalist diet (Gibbs et al., 2011, 2013; Lyons et al., 2020). Geographic differences in diet preferences have been observed in the eastern massasauga (Weatherhead et al., 2009), and other massasauga species have been classified as dietary generalists (Holycross & Mackessy, 2002). With such limited information available on eastern massasauga diet, more accurate and minimally invasive means of identifying the prey species they consume are necessary to further understand their ecology and feeding preferences, and whether resource limitation may be contributing to their declines.

Our study is the first to apply a DNA metabarcoding approach to identify what prey species eastern massasaugas are mainly consuming in populations distributed throughout the lower peninsula of Michigan. In addition, we compare diets between individuals to provide further insight as to whether feeding preferences differ between age classes, seasons, and populations. We hypothesize that the bulk of eastern massasauga diet will consist of a range of mammal species, but that individuals will also feed opportunistically on other taxa such as snakes and birds. Furthermore, we outline an approach in this study that is broadly applicable to future dietary studies of any reptile species. To our knowledge, this study is the first to apply a DNA metabarcoding diet analysis to a rattlesnake species, and the first to use a molecular method to analyze eastern massasauga diet.

## 2 METHODOLOGY

### 2.1 Sample collection and preservation

**FIGURE 2:**
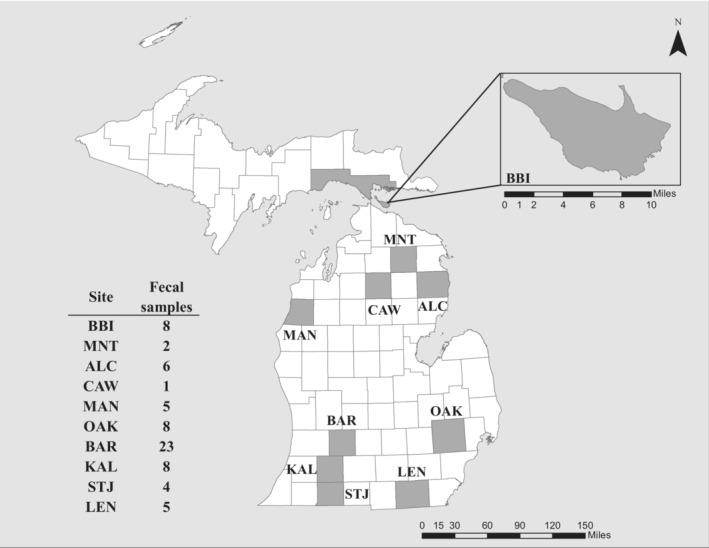
Map of eastern massasauga sampling locations. Ten sites were sampled, all in the lower peninsula of Michigan, USA, except for Bois Blanc Island (BBI; located off the northern coast of the lower peninsula). Site names indicate the county they were collected from (BBI = Bois Blanc Island, MNT = Montmorency, ALC = Alcona, CAW = Crawford, MAN = Manistee, OAK = Oakland, BAR = Barry, KAL = Kalamazoo, STJ = St. Joseph, LEN = Lenawee). Numbers indicate fecal samples representing each site after the final steps of sequence filtering. Specific site locality is withheld and labeled at county level to prevent illegal collection.

We carried out visual encounter surveys from May–September in 2018 and 2019 during the eastern massasauga active season (approximately April to October; Szymanski et al., 2015). To get an accurate representation of massasauga diet throughout the lower peninsula of Michigan, we selected sites that were distributed across the state (Figure 2). Specific site locality below the county level is withheld to prevent illegal collection. In addition, we collected samples from Bois Blanc Island (BBI), located off the northern coast of the lower peninsula. Considering the possibility that diet shifts may occur throughout the season, we visited each site multiple times during the active season whenever possible. When a snake was opportunistically located, it was captured using tongs, and safely secured in a cloth bag. All capture locations were recorded using handheld GPS units.

Snakes were secured in a clear plastic tube for safe handling. Newly captured individuals were marked using a passive integrated transponder (PIT) tag for permanent identification. Fecal material was directly extracted from the snake by placing its tail into a 50 mL conical tube and gently palpating until defecation occurred. All newly captured snakes and snakes that were recaptured more than 2 weeks apart were palpated, but not all captures produced samples. For each snake, we measured mass (to the nearest g), snout‐vent length (SVL) (cm), and tail length (cm). Sex was determined by probing of the cloaca, and gravidity was determined for adult females by gentle palpation. Collection attempts of fecal samples from gravid females were restricted to earlier in the active season (May‐early July), while gravid females with well‐developed embryos were limited to opportunistic collection. If snakes were unable to be probed due to small size, we determined sex based on the subcaudal scute count (≥25 subcaudals were considered male). Individuals were classified as adult, juvenile, or young (snakes born the previous year) based on SVL measurements. Females with SVL ≥45 cm and males ≥43 cm were classified as adults (Bradke et al., 2018); and snakes of both sexes with SVL ≥30 cm were classified as juveniles. Snakes with SVL < 30 cm that possessed one or fewer rattle segments, followed by a single complete terminal rattle segment (without breakage) were considered young. Following processing, each massasauga was returned to its capture site.

Samples collected in the summer of 2018 were temporarily placed in a cooler containing ice following collection and moved to long‐term storage at −80°C as soon as possible. Due to field conditions, the length of time these samples were stored on ice greatly varied from a few hours to a few days until a freezer was accessible. In the following field season (May–August 2019), samples were immediately frozen using a dry ice‐ethanol bath. Each sample collection tube remained in the bath for a few minutes until the sample was frozen. These samples were then preserved in a cooler with dry ice, where they remained frozen until being moved to a −80°C freezer for long‐term storage. In total, 102 samples were collected across 10 populations (Figure 2).

### 2.2 DNA extraction

Extraction from each fecal sample was carried out using QIAamp PowerFecal DNA Kits (Qiagen) following the standard protocol requiring 0.25 g of stool. A random subsample was taken if the sample mass exceeded 0.25 g, while the entire sample was used if it was less than 0.25 g. DNA extractions took place in a laminar flow hood with UV sterilization to prevent contamination. Snakes excrete all wastes from their cloaca; therefore, urates were sometimes present as a yellow solid in fecal samples. As dietary information cannot be obtained from urates, we avoided including them to the best of our ability during the extraction process. One negative control using double‐distilled water containing only reagents was included during each extraction batch to test for contamination. Extraction success was confirmed by gel electrophoresis on a 1.5% agarose gel. Although we collected 102 fecal samples in total, sufficient DNA for amplicon sequencing was obtained from 83 samples. A random subset of extracted DNA samples was quantified using a NanoDrop™ OneC Spectrophotometer (Thermo Fisher Scientific Inc.) with three replicates per sample. The quantity of DNA per sample ranged from 5.4–51.4 ng/μL (24.7 ng/μL on average), which we used to determine a suitable volume of DNA for PCR reactions (see Prey Amplification section).

### 2.3 Primer selection

To identify all potential prey, we selected the universal metazoan forward mlCOIintF (Leray et al., 2013) and reverse jgHCO2198 (Geller et al., 2013) primer set, targeting a 313 bp fragment of the cytochrome oxidase subunit 1 (CO1 region; see Table 2 for the list of primers used in this study). This primer pair is designed to amplify all metazoan taxa and is commonly used in DNA metabarcoding dietary assessments (e.g., Bohmann et al., 2018; Milner et al., 2021; Robeson et al., 2018). The reverse primer jgHCO2198 is a redesign of the Folmer reverse primer HCO2198 (Folmer et al., 1994), corrected for mismatches and with increased degeneracy to allow for broader taxonomic amplification (Geller et al., 2013). Additional Illumina index‐specific overhangs were added onto the 5′ ends of the forward and reverse primers.

**TABLE 2:** Forward, reverse, and blocking primer sequences used in this study.

Primer name	Sequence 5′‐3′	Source
mlCOIintF (Forward)	GGWACWGGWTGAACWGTWTAYCCYCC	Leray et al. (2013)
jgHCO2198 (Reverse)	TAIACYTCIGGRTGICCRAARAAYCA	Geller et al. (2013)
EMR_mlCOIintF_BLK	TTTATCCCCCCCTCTCCGGAAATCTAGTC‐3InvdT	This study

*Note*: The blocking primer was designed based on the mlCOIintF forward primer. It overlaps 10 bp at the 3′ end of the forward primer and extends 19 bp into the massasauga‐specific sequence. The inverted dT at the 3′ end of the blocking primer halts the polymerase and prevents amplification of the host (massasauga) DNA.

### 2.4 Predator blocking oligonucleotide design

When using universal primers, the nontarget (predator) DNA will amplify at a larger scale and limit successful amplification of the prey DNA due to the degraded nature of the prey DNA. To increase the chances of identifying rare prey items, we designed an annealing inhibiting blocking oligonucleotide (Vestheim & Jarman, 2008). To design the blocking primer, eastern massasauga‐specific sequences along with available sequences of previously recorded prey items from past diet studies (Holycross & Mackessy, 2002; Keenlyne & Beer, 1973; Shepard et al., 2004; Tetzlaff et al., 2015; Weatherhead et al., 2009) were downloaded from GenBank (Table S1). Eastern massasaugas have been documented to consume frogs (Hallock, 1991; Ruthven, 1911) and other snakes (Hallock, 1991; Keenlyne & Beer, 1973; Ruthven, 1911; Tetzlaff et al., 2015; Weatherhead et al., 2009), but these have not always been identified to species, so geographically relevant species were also considered potential prey and downloaded from GenBank. Eastern massasauga, potential prey, and the forward mlCOIintF primer were aligned using ClustalW in MEGA X (Kumar et al., 2018). A region of variability between eastern massasauga and potential prey was visually identified as a suitable location to place the 3′ end of the blocking primer. We aimed to prevent potential snake prey from being inadvertently blocked. However, there were often only a few mismatches as the COI region is highly conserved (Table S2). We designed the blocking primer to overlap 10 bp at the 3′ end of the mlCOIintF forward primer and extend 19 bp into the massasauga‐specific sequence (Table 2). Blocking oligonucleotides for diet studies are typically designed using a C3 spacer modification on the 3′ end to prevent amplification (Vestheim & Jarman, 2008). However, we were unable to consistently block eastern massasauga DNA using this modification, likely due to the 3′–5′ exonuclease activity of the high‐fidelity polymerase degrading the C3 Spacer. We instead opted for a stronger 3′ inverted dT modification. To test the specificity of the blocking primer, we performed PCR (see prey amplification section below for cycle conditions) on three mammal specimens (shrew, vole, and mouse), one sample containing pure eastern massasauga DNA, and one eastern massasauga fecal sample to be used for downstream analyses. We determined the blocking primer as suitable when the band of amplified pure eastern massasauga DNA was notably lighter compared to the PCR reactions containing potential prey DNA and eastern massasauga fecal DNA (Figure S1).

### 2.5 Prey amplification

To limit errors while generating amplicons during amplification, we selected the NEBNext® Q5U® Master Mix (New England Biolabs, USA) high‐fidelity polymerase that is compatible with the inosine bases present in the jgHCO2198 reverse primer and possesses a 3′‐5′ exonuclease activity. The annealing inhibiting blocking primer was included at 15× the concentration of the universal primers. PCR was carried out using the following conditions: 12.5 μL of NEBNext Q5U Master Mix for a final 1× concentration, 3 μL of genomic DNA, 1.25 μL of the forward and reverse primer (0.5 μM final concentration), 1.875 μL blocking oligonucleotide (7.5 μM final concentration), and 5.125 μL of nuclease‐free water (NEB) for a 25 μL total reaction volume. We carried out an initial denaturation at 98°C for 30 s followed by 30 cycles: denaturation at 98°C for 10 s, annealing at 64°C for 30 s, extension at 72°C for 60 s followed by a final extension at 72°C for 5 min. PCR amplification success was confirmed by gel electrophoresis on a 1.5% agarose gel. The extraction negative controls were included during the PCR steps to check for contamination and no discernable PCR product appeared by gel electrophoresis.

### 2.6 Library preparation and sequencing

To remove primer dimers after the initial PCR reaction, 25 μL of PCR product was cleaned using AMPure XP beads (Beckman Coulter). Amplicons were indexed using Nextera XT indexes (Illumina) using the following cycling conditions: 95°C for 3 min, followed by 8 cycles of 95°C for 30 s, 55°C for 30 s, 72°C for 30 s, and a final step of 72°C for 5 min. Indexed amplicons were again purified using AMPure XP beads. Purified libraries were quantified using a Qubit Fluorometer (ThermoFisher Scientific), and the average fragment size was determined using an Agilent 2100 Bioanalyzer. In total, 83 samples were prepared for sequencing. Libraries were then normalized at equal molarities (4 μmol/L) and pooled. The pooled libraries were denatured using 0.2 N NaOH and diluted to 10 pmol/L. We added a 15% spike‐in of 10 pmol/L Phi‐X due to the expected low diversity of the sample libraries. The pooled libraries and Phi‐X were sequenced on an Illumina MiSeq Sequencer using a v3 600‐cycle cartridge for 2 × 300 bp paired‐end read sequencing.

### 2.7 Sequence processing and taxonomic classification

All sequence processing and taxonomic classification were carried out using the program QIIME 2 v.2020.11 (Bolyen et al., 2019). The Cutadapt plugin (Martin, 2011) was used to trim the forward and reverse primers from the demultiplexed sequences using the cutadapt trim‐paired command with the following parameters: ‐‐p‐match‐adapter‐wildcards, ‐‐p‐match‐read‐wildcards to allow matching of IUPAC wildcards, ‐‐p‐discard‐untrimmed to discard any reads in which the primers were not found, and the default ‐‐p‐error‐rate 0.1. The lengths to truncate the forward and reverse reads were based on sequence quality plots following trimming. We used DADA2 (Callahan et al., 2016) to truncate and denoise the trimmed sequences into amplicon sequence variants (ASVs), which corrects for amplicon errors from the sequencing run. Compared to OTUs, ASVs are distinct biological sequences providing more precise taxonomic identification, while such diversity can be missed by OTU clustering (Callahan et al., 2017). While ASVs have yet to be heavily adapted into dietary studies, this denoising method has been found to outperform OTU clustering with mock dietary datasets (O'Rourke et al., 2020). Following the denoising step, paired end reads were merged, and chimeric and singletons were identified and removed. To perform taxonomic classification, we used the MIDORI_UNIQ_GB240_CO1 database (Machida et al., 2017) consisting of unique sequences for all eukaryotes available in the GenBank 240 release (October 2020). We first attempted taxonomic classification using classify‐sklearn (Pedregosa et al., 2011) with a kmer‐based Naive Bayes‐trained classifier. However, this classified method resulted in many ambiguous taxa along with taxa that did not fit the sampled geographic range. We instead opted for an alignment approach using the BLAST+ plugin (Camacho et al., 2009). This performs local alignments between the reference reads and query sequences and performs least common ancestor (LCA) classification. We used the classify‐consensus‐blast command for taxonomic classification with the following parameters: ‐‐p‐maxaccepts 1000 as the maximum number of hits to keep for each query, ‐‐p‐perc‐identity 0.97 as the minimum percentage that the query sequence should match the reference sequence, ‐‐p‐query‐cov 0.89 as the percentage of the sequence to be aligned to the reference database, and –p‐strand both to align the forward and reverse query sequences to the reference sequences (O'Rourke et al., 2020).

Following classification, we filtered out unassigned sequences, taxonomy that did not have a phylum level identification, or any eastern massasauga or human contaminant using the qiime taxa filter‐table and filter‐seqs commands. Environmental contaminants such as bacteria and fungi were also filtered. Taxa that we deemed to be environmental contaminants or unlikely prey items were also filtered (see Results).

### 2.8 Statistical analyses

Sequence counts are often not an accurate reflection of the overall abundance of prey taxa consumed due to biases including different digestion rates, DNA density, and primer and sequencing bias (Deagle et al., 2013). Therefore, we only relied on presence/absence data for our analyses. We calculated %FOO (frequency of occurrence) for each prey species as the total number of times each species appeared across individual samples averaged over the total number of samples following filtering steps (*n* = 70). To determine if we captured the full dietary diversity in our dataset, a species accumulation curve of the presence/absence data was calculated in R (R Core Team, 2021) v.4.0.5. Species accumulation curves display the number of taxa that are detected within a dataset as the number of samples accumulates. The accumulation curve was generated using the *specaccum* function in the vegan package (v.2.5‐7, Oksanen et al., 2020) and the random method to add the fecal samples in random order.

To determine the differences in diets between age classes, seasons, and populations, a nonmetric multidimensional scaling (NMDS) ordination was generated in a Jaccard matrix with 999 permutations with the *vegdist* function. A Permutational Multivariate Analysis of Variance (PERMANOVA) post‐hoc test with 999 permutations was run for each separate analysis (age class, population, and sex) using the *adonis2* function in the vegan package (v.2.5‐7, Oksanen et al., 2020). If a significant *p*‐value was obtained, we then ran a pairwise PERMANOVA using the function *pairwise.adonis2* in the pairwise Adonis package (v.0.4; Martínez Arbizu, 2020) with 999 permutations and a Jaccard matrix to determine which variables were statistically different. *p*‐Values were Holm‐corrected to account for multiple comparisons. To identify which species drove any significant differences, we ran a similarity percentage (SIMPER) test in the vegan package with 999 permutations. Several prey taxa were only consumed once by one individual; therefore, we limited the taxa included in the NMDS analysis to only prey with more than one occurrence (*Blarina brevicauda*, *Sorex cinereus*, *Condylura cristata*, *Microtus pennsylvanicus*, *Peromyscus leucopus*, *Napaeozapus insignis*, and *Zapus hudsonius*) across all samples to allow convergence, therefore, removing three samples from these analyses (*n* = 67). When site differences were compared, Crawford County (CAW) was removed as it only contained one sample (*n* = 66).

## 3 RESULTS

After sequencing the 83 total samples, we obtained 6,016,360 raw sequence reads prior to any filtering steps. Read counts per sample ranged from 8461 to 154,512, with a median of 69,913 and mean of 72,486 reads per sample. In total, the DADA2 pipeline in QIIME2 identified 6102 ASVs belonging to 164 different taxa. There were 707,306 sequences identified as eastern massasauga, meaning the host DNA comprised 11.8% of sequences before filtering, and 13,109 or 0.2% of sequences belonged to human contaminant DNA. After filtering out unassigned taxa, taxa without a phylum level identification, eastern massasauga, and nonprey items (algae, fungi, etc.), DADA2 identified 73 ASVs belonging to 12 metazoan prey taxa. During the filtering process, 13 samples were removed that did not detect any prey DNA, resulting in 70 samples that contained prey. Forty‐six of the samples came from adult snakes, 13 from juveniles, and 11 from young snakes.

We detected a number of metazoan taxa that were unlikely prey items and determined these taxa to be environmental contaminants or a result of secondary predation (i.e., the target prey had consumed it prior to being eaten by the snake); therefore, they were filtered out and excluded from subsequent dietary analyses. These species included larger mammals such as domestic dog (*Canis lupus familiaris*), domestic cat (*Felis catus*), white‐tailed deer (*Odocoileus virginianus*), multiple earthworm species (*Dendrodrilus rubidus*, *Lumbricus rubellus*, *Lumbricus terrestris*), and a snail (*Oxyloma verrilli*). We also detected numerous arthropod species (mites, ticks, etc.) that were present in small frequencies and represented by very low sequence counts (Tables S3 and S4). Multiple parasitic species were detected, including nematodes in the Rhabditida order (*Crossonema menzeli*, *Caenorhabditis remanei,* and *Baylisascaris procyonis*). Additionally, the parasitic Apicomplexa; *Caryospora bigenetica* was detected in nearly every individual (Table S4). All the species detected in the eastern massasauga fecal samples are presented in the supplementary files (Tables S3 and S4).

**FIGURE 3:**
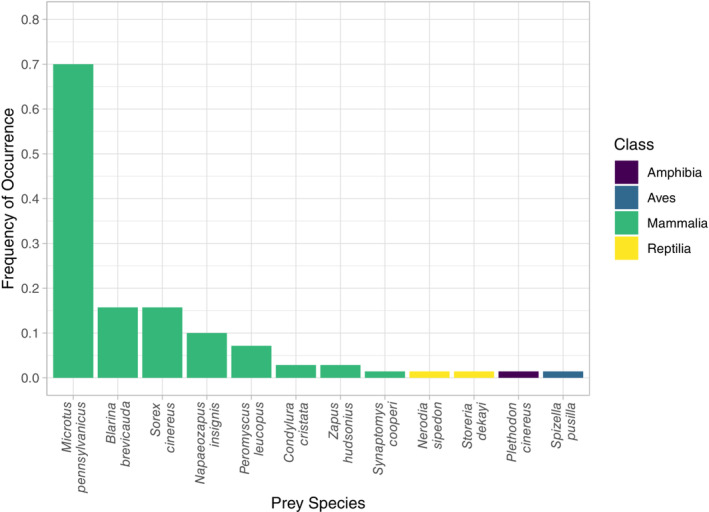
Frequency of occurrence (FOO) of all the prey items identified in our dataset down to the species level. FOO calculations were carried out using the presence/absence occurrences of prey averaged across all samples.

The number of prey species detected per sample ranged from 1–3 taxa. Frequency of occurrence (%FOO) data showed that small mammal species were the prey category most frequently consumed (Figure 3 and Table 3). Specifically, the meadow vole (*Microtus pennsylvanicus*) was the most common prey (70%) of the eastern massasauga. The northern short‐tailed shrew (*Blarina brevicauda*) and masked shrew (*Sorex cinereus*) each made up 15.7% of eastern massasauga diet, followed by the woodland jumping mouse (*Napaeozapus insignis*; 10%), the white‐footed mouse (*Peromyscus leucopus*; 7.1%), star‐nosed mole (*Condylura cristata*; 2.9%), and meadow jumping mouse (*Zapus hudsonius*; 2.9%). Two reptile prey were detected, with one occurrence of a northern water snake (*Nerodia sipedon*; 1.4%), and Dekay's brown snake (*Storeria dekayi*; 1.4%) in two different individuals. Additionally, there was one occurrence each (1.4%) of a red‐backed salamander (*Plethodon cinereus*), southern bog lemming (*Synaptomys cooperi*), and field sparrow (*Spizella pusilla*; Figure 3).

**TABLE 3:** Number of detections and percent frequency of occurrence (%FOO) of eastern massasauga rattlesnake prey for young, juvenile, and adult age classes.

Species name	Common name	Young consumers	%FOO young	Juvenile consumers	%FOO juvenile	Adult consumers	%FOO adult	Total consumers	%FOO total
*Microtus pennsylvanicus*	Meadow vole	8	72.73	11	84.62	30	65.22	49	70.00
*Blarina brevicauda*	Northern short‐tailed shrew	1	9.09	1	7.69	9	19.57	11	15.71
*Sorex cinereus*	Masked shrew	5	45.45	4	30.77	2	4.35	11	15.71
*Napaeozapus insignis*	Woodland jumping mouse	1	9.09	1	7.69	5	10.87	7	10.00
*Peromyscus leucopus*	White‐footed mouse	1	9.09	0	0	4	8.70	5	7.14
*Condylura cristata*	Star‐nosed mole	0	0	0	0	2	4.35	2	2.86
*Zapus hudsonius*	Meadow jumping mouse	0	0	0	0	2	4.35	2	2.86
*Plethodon cinereus*	Red‐backed salamander	0	0	0	0	1	2.17	1	1.43
*Spizella pusilla*	Field sparrow	0	0	0	0	1	2.17	1	1.43
*Nerodia sipedon*	Water snake	0	0	0	0	1	2.17	1	1.43
*Storeria dekayi*	Brown snake	0	0	0	0	1	2.17	1	1.43
*Synaptomys cooperi*	Southern bog lemming	0	0	0	0	1	2.17	1	1.43

*Note*: %FOO was calculated by dividing the number of detections over the total number of samples for each age class, and the total includes all age classes.

All age classes consumed several mammal species, with the meadow vole being the most commonly consumed for young, juvenile, and adults (72.7%, 84.6%, and 65.2%, respectively). Adults consumed the widest breadth of prey, consuming all 12 prey species detected in this study, while juveniles consumed four and young snakes consumed five different prey species (Table 3). Following meadow voles, adults most often ate northern short‐tailed shrews (19.6%), woodland jumping mice (10.9%), and white‐footed mice (8.7%). Adult snakes were the only age class to consume the star‐nosed mole, meadow jumping mouse, and southern bog lemming. Furthermore, only adult snakes preyed upon bird (field sparrow), amphibian (red‐backed salamander), or snake (brown snake and water snake) species. Masked shrews were the second most common prey items for young (45.5%) and juveniles (30.8%), while only making up 4.3% of adult diets (Table 3). When comparing only prey consumed more than once, the PERMANOVA did not detect a significant difference in diets between young, juvenile, and adult individuals (*p* = .076; Figure S2). There were also no significant differences in prey items based on sex or month.

The number of prey taxa detected at each site ranged from two to eight different species. The PERMANOVA identified a significant difference in the composition of prey species consumed among the nine sites (*p* = .035). The pairwise PERMANOVA further revealed that the Lenawee County site significantly differs from Barry County (*p* = .021), Kalamazoo (*p* = .037), and Bois Blanc Island (*p* = .029). Montmorency County significantly differed from Kalamazoo (*p* = .024) and Barry County (*p* = .046). Results of the SIMPER analysis indicated that northern short‐tailed shrews and eastern meadow voles were mainly responsible for driving the site differences. Northern short‐tailed shrews were more commonly consumed by the population in Lenawee County, whereas eastern meadow voles were more common in Kalamazoo and Barry County.

## 4 DISCUSSION

Our results illustrate that DNA metabarcoding approaches are a robust, efficient way to assess snake diets. Despite the degraded nature of the DNA in fecal samples, we consistently identified several prey items, along with nontarget items as well (e.g., fungi, algae, and parasites). Our metabarcoding results demonstrate that eastern massasaugas strongly prefer small mammal prey, yet individuals occasionally consume other prey including amphibians, reptiles, and birds (Figure 3). Our metabarcoding approach identified prey species that had been previously documented in traditional eastern massasauga diet studies (Table 1) in addition to multiple new prey items that were not previously documented. New prey included the southern bog lemming, star‐nosed mole, northern water snake, red‐backed salamander, and field sparrow (Figure 3). Identifying previously documented prey (Table 1) along with numerous new prey taxa demonstrates that metabarcoding techniques are reliable and have a higher resolution. This, along with the less invasive nature of sample collection for metabarcoding approaches, make it a more favorable method compared to traditional approaches.

**FIGURE 4:**
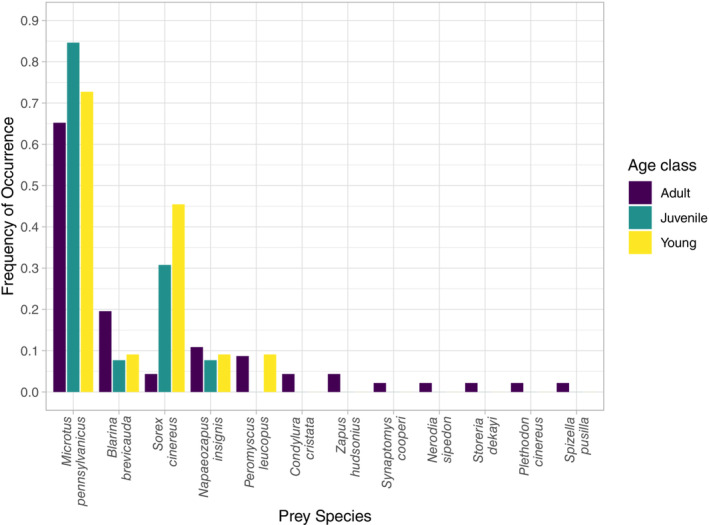
Frequency of occurrence (FOO) of all the prey items identified in our dataset down to the species level grouped by adult, juvenile, and young age classes. FOO calculations were carried out using the presence/absence occurrences averaged across the number of samples for each age class category.

All age classes consumed multiple mammal species, the most common being the meadow vole. Young and juvenile individuals tend to have a more limited prey base compared to adult individuals, with the younger age classes feeding mostly on meadow voles (young 73%, juvenile 85%), masked shrews (young 46%, juvenile 31%), and northern short‐tailed shrews (young 9%, juvenile 8%; Figure 4). Young and juvenile snakes each consumed four and five different species, respectively, while adults consumed 12 species. Previous eastern massasauga dietary studies have suggested evidence of an ontogenetic diet shift occurring, with the younger snakes being the main consumer of other snake species (Keenlyne & Beer, 1973). However, we only identified two other snake species in our dietary dataset, with both species consumed by adult massasaugas (Figure 4) and did not find evidence of smaller snakes preferring other snake prey. The PERMANOVA did not detect a significant difference between age classes, but this is likely due to the differences in sample sizes among age classes as younger snakes are rarer to come across during surveys. Similar to the juvenile prey items identified in our study, Shepard et al. (2004) found wild neonate individuals had largely consumed southern short‐tailed shrews (*Blarina carolinensis*) via stomach content regurgitation in an Illinois population. Compared to *Microtus*, *Peromyscus*, and *Napaeozapus* species, masked shrews and northern short‐tailed shrews are among the smallest mammals observed in our dietary dataset, with maximum adult body lengths around 10 and 14 cm, respectively (Kurta, 2017). It is important to note that determining the age class of the consumed prey is not feasible with metabarcoding techniques. Snakes are gape‐limited predators, and it is likely that the larger mammals consumed by smaller snakes, such as meadow voles, were younger individuals. Consistent with a gape‐limited predator, massasaugas appear to be more likely to consume smaller mammal prey during their first few years, later moving on to a larger, more generalized mammal diet during adulthood.

Eastern massasaugas appear to be somewhat opportunistic predators. The sampled populations heavily preyed on mammals, with small differences in the prey species consumed in different populations. Smaller individuals (young and juveniles) seemed to focus their diet on mammals with smaller body sizes such as shrews. Adult individuals consumed a more diverse number of prey species while retaining the small shrew species in their diet (Figure 4 and Table 3). These findings suggest the snakes may consume what prey is most abundant and readily available in that habitat, similar to previous massasauga diet studies (Keenlyne & Beer, 1973; Weatherhead et al., 2009). Opportunistic feeding behavior is beneficial for these snakes from a conservation perspective, in that they will have food available if potential prey species (small mammals) are abundant. Due to gape limitations, smaller snakes during their first few years of growth are most limited by prey abundance such as juvenile voles, or smaller shrews. In order to have adequate food sources available for all massasauga age classes, small mammal populations need to be healthy and diverse. Therefore, we suggest the maintenance of small mammal populations should be considered when conservation strategies are developed for eastern massasaugas.

When eastern massasauga diet was assessed in Ontario and Ohio populations by fecal sample dissection, the bulk of their diet consisted of mammals with the occasional snake prey (Weatherhead et al., 2009). However, chipmunks (*Tamias striatus*) were classified as the most common prey item in both Ontario and Ohio populations by identification of hair samples from feces (Weatherhead et al., 2009). We did not identify any mammal species as large as chipmunks or squirrels using our metabarcoding approach. The discrepancies here could result from real differences in diet among these populations or they could be a result of the methodological differences between traditional and metabarcoding techniques. Moreover, the species we identified in our dataset are more consistent with being wetland species. Dissection of fecal samples may leave important prey items overlooked or may result in morphological misidentification of prey when using remains such as hair, whereas metabarcoding diet characterization could decrease the chance of a false positive. When traditional and metabarcoding approaches were compared when studying the diet of the Selvagens gecko, the traditional methods resulted in overlooked diet items that were only identified through metabarcoding (Gil et al., 2020). We encourage future studies focusing on snake diet to take the differences in sensitivity between traditional and metabarcoding methods observed here into consideration.

Geographically, all the identified prey ranges overlap with the eastern massasauga range. However, there were five occurrences of woodland jumping mice that occurred outside of that species' current recorded distribution. We aligned the reference sequences of closely related species in the range, and the sequences classified as woodland jumping mouse in our dataset were blasted in NCBI to confirm identification. Woodland jumping mice are historically distributed in the northern lower peninsula (Kurta, 2017; Myers et al., 2009). Our prey occurrences were located in Barry, Kalamazoo, and Lenawee County, further south than their current recorded distribution. Additionally, we detected woodland jumping mice on Bois Blanc Island, in which there is limited knowledge on the mammal species composition (Myers et al., 2009). There are currently no records of recent shifts in distribution to the southern lower peninsula; however, recent surveys have primarily focused on the northern region of the lower peninsula (Myers et al., 2009). Considering that we detected woodland jumping mice outside of their known range in independent fecal samples, these observations could also provide insight into the current distribution for these prey species. For example, in addition to detecting woodland jumping mice on Bois Blanc Island, northern short‐tailed shrews and meadow voles were identified in the diets of individuals from this location. These detections can provide insight on what species are present when traditional surveys have not taken place in sites such as Bois Blanc Island. Predators in diet metabarcoding studies have been referred to as “biodiversity capsules” (Boyer et al., 2015; Nørgaard et al., 2021) by providing information on the species composition in the same habitat. With the bulk of eastern massasauga diet consisting of several small mammal species, our metabarcoding results could complement field observations, and perhaps help overcome the limitations of detection with small mammal trapping techniques.

A variety of nontarget taxa were identified from eastern massasauga fecal samples along with the prey items. DNA metabarcoding techniques are incapable of differentiating between direct and secondary consumption, or accidental consumption of material that may occur during prey capture. The invertebrate taxa we identified consisted of small taxa such as earthworms, moths, flies, and ants (Tables S3 and S4), and these were removed from the analysis since we could not confidently label them as prey items. Eastern massasaugas are venomous ambush predators, and it is unlikely the small insect and arachnid occurrences are a result of direct consumption. Many of the mammals detected in our dataset are invertebrate specialists. Shrews, voles, and mice commonly feed on small insects and arachnids. Land snails and earthworms are also documented prey of numerous mammal species we identified in our dataset, including star‐nosed moles and northern short‐tailed shrews (Kurta, 2017). In addition, invertebrates were most often detected along with mammal prey, with lower read counts compared to the mammals when detected in the same sample (Table S4). Due to these reasons, we determined these taxa to mainly represent secondary prey occurrences and choose to filter out all invertebrate species. We acknowledge that a snake may have consumed an invertebrate directly that would not be detected after filtering; however, we did not want to overrepresent the ecological importance of a species. We also detected several larger mammalian species that are likely to be secondary detections or environmental contamination instead of prey items, including white‐tailed deer (*Odocoileus virginianus*). One possible explanation for this could be due to scavenging events from the consumed small mammal species. For example, small mammals such as *Peromyscus* and *Blarina* spp. have been observed to scavenge white‐tailed deer carcasses (Jennelle et al., 2009). The presence of these larger mammals could also be a result of bloodmeals from the ectoparasites we detected, such as ticks, chiggers, and mites that were then consumed by small mammal prey. Environmental contamination should also be considered as a possible source, as detection of these larger mammals could potentially be due to water intake or from the snake moving throughout its habitat. The detection of numerous species due to secondary consumption and environmental contamination shows how sensitive metabarcoding techniques are, and that results for diet studies should be carefully considered to avoid over‐estimating the breadth of a predator's diet. Furthermore, to avoid contamination due to the high sensitivity, caution should be taken during sample collection and laboratory work to avoid false positive detections of diet items.

We identified three nematode species (*Crossonema menzeli*, *Caenorhabditis remanei*, and *Baylisascaris procyonis*) in 10 diet samples, suggesting the potential of intestinal parasites in some of these populations. Numerous parasitic nematodes infect a variety of snake species (Bursey & Brooks, 2011; Hallinger et al., 2020; Lettoof et al., 2020), including the eastern massasauga (Hallock, 1991). However, like secondary prey items, some of these parasites may be infecting the primary prey species and not associated with the sampled massasauga rattlesnakes. *B. procyonis* is a raccoon parasite also found in small mammals that act as intermediate hosts (Kazacos, 2016), but reptiles are reported to not be susceptible to infection (Kazacos, 2016; Weinstein et al., 2018). Snakes with poor body conditions have been noted to possess a larger number of parasites (Hallinger et al., 2020); however, all the snakes we identified appeared healthy, and had no signs of snake fungal disease. The detection of *B. procyonis* is likely due to the large number of small mammals consumed.

While our use of metabarcoding for diet analysis provides important information regarding the array of prey items consumed by both adult and juvenile massasauga rattlesnakes, there are a few caveats that must be taken into consideration. Rattlesnakes, including eastern massasaugas (Hallock, 1991; Ruthven, 1911), do occasionally exhibit cannibalism (Mociño‐Deloya et al., 2009; Prival et al., 2002). If cannibalism had occurred in any of our collected samples, we would be unable to identify it as a prey item due to DNA metabarcoding limitations associated with the potential predator DNA co‐amplification. Cannibalism cannot be excluded as a possibility for this species and may have occurred in the sampled massasauga populations. The eastern massasauga‐specific blocking primer is designed to compete with the universal primers and limit the amplification of the predator DNA. Although the blocking primer was effective in preventing the predator DNA from swamping out prey species, it did not completely block out predator DNA considering eastern massasauga DNA compromised 11.8% of the total sequences. Blocking primers primarily limit amplification of the nontarget DNA, rather than completely halting amplification (Vestheim & Jarman, 2008). Although we opted for the stronger 3′ inverted dT blocking modification, the exonuclease activity of the high‐fidelity polymerase likely still degraded the modification, thereby decreasing the effectiveness of the blocking primer. However, we believe the increased accuracy of the high‐fidelity polymerase allowed closely related prey to be discerned during sequencing and outweighed the benefits of the blocking primer working at full efficiency. Future studies should take this into consideration when developing a blocking primer and selecting an appropriate polymerase. It should also be noted that blocking primers may also block amplification of target prey DNA if they are closely related to the predator (Piñol et al., 2015; Shehzad et al., 2012). Since the CO1 region is highly conserved, it can be challenging to find a suitable sequence specific enough to the predator DNA without also blocking closely related prey. Although we detected two snake species, the use of the blocking primer may have inadvertently blocked the amplification of other closely related snake species (Table S2). This is particularly relevant considering reptile species can be underrepresented in CO1 barcoding databases (Vences et al., 2012). Therefore, it is possible that potential reptile prey may be overlooked using metabarcoding approaches with blocking primers to study snake diet. We suggest that adding known reptile sequences to barcoding databases be prioritized to improve the success of future dietary studies with a reptile host and/or potential reptile prey. Finally, we note that our species accumulation curve did not plateau, suggesting that we may not have captured the full diversity of eastern massasauga rattlesnake diet (Figure S3). DNA metabarcoding is highly sensitive and captures only a snapshot in time. As we observed massasaugas to be opportunistic feeders, there were multiple occurrences of only one detection of a prey species (Figure 3 and Table 3). A larger sample size across the entire active season may be required from each site to fully capture the diversity of prey consumed by eastern massasauga rattlesnakes.

We conclude that DNA metabarcoding of feces is a reliable way to characterize snake diet. Our results complement and expand upon previous characterizations of eastern massasauga diet, and further demonstrate that small mammals constitute the majority of prey for these snakes. From a conservation perspective, the opportunistic feeding preferences we identified are beneficial for the long‐term survival of eastern massasugas. We suggest that the abundance of small mammal populations be taken into consideration when developing management plans for the eastern massasauga rattlesnake. This study is one of few that has successfully characterized the diet of a snake species using metabarcoding. We have demonstrated the success of using this minimally invasive method to study the diet of threatened reptiles, which can be used as a guide in future studies.

## AUTHOR CONTRIBUTIONS

**Alyssa Swinehart:** Conceptualization (equal); data curation (lead); formal analysis (lead); investigation (lead); methodology (lead); resources (lead); visualization (lead); writing – original draft (lead); writing – review and editing (lead). **Charlyn Partridge:** Conceptualization (supporting); methodology (supporting); resources (supporting); writing – review and editing (supporting). **Amy Russell:** Conceptualization (supporting); methodology (supporting); writing – review and editing (supporting). **Arin Thacker:** Resources (equal). **Jennifer Kovach:** Resources (equal). **Jennifer Moore:** Conceptualization (equal); funding acquisition (lead); methodology (supporting); project administration (equal); resources (supporting); supervision (lead); writing – review and editing (supporting).

## Supporting information


Appendix S1.

Appendix S2.

Appendix S3.

## Data Availability

Raw sequence reads are uploaded into the NCBI SRA (sequence read archive), accession number: PRJNA947179. Metadata and bioinformatic code are available on Dryad: https://doi.org/10.5061/dryad.rv15dv4c9.
