# Supplementary material for: Diet of a threatened rattlesnake (eastern massasauga) revealed by DNA metabarcoding
Source: Ecol Evol. 2023 Apr 26;13(4):e10029. doi: 10.1002/ece3.10029 (PMC10131807; doi:10.1002/ece3.10029)
Supplement: Supplementary file 1 — Appendix S1. [file ECE3-13-e10029-s001.docx]

**Supplemental Information for:**

**Diet of a threatened rattlesnake (eastern massasauga) revealed by DNA metabarcoding.**

Alyssa Swinehart, Charlyn Partridge, Amy Russell, Arin Thacker, Jennifer Kovach, Jennifer Moore

Figure S1. PCR amplifications with the blocking oligonucleotide EMR_mlCOIintF_BLK included. The first 3 wells are amplified potential mammal prey. The EMR well shows pure eastern massasauga rattlesnake DNA used as a template, and the last well is amplification of an eastern massasauga fecal sample. Note that due to primer size and tags added for future sequencing, the product size appears larger than the target 313 bp. **
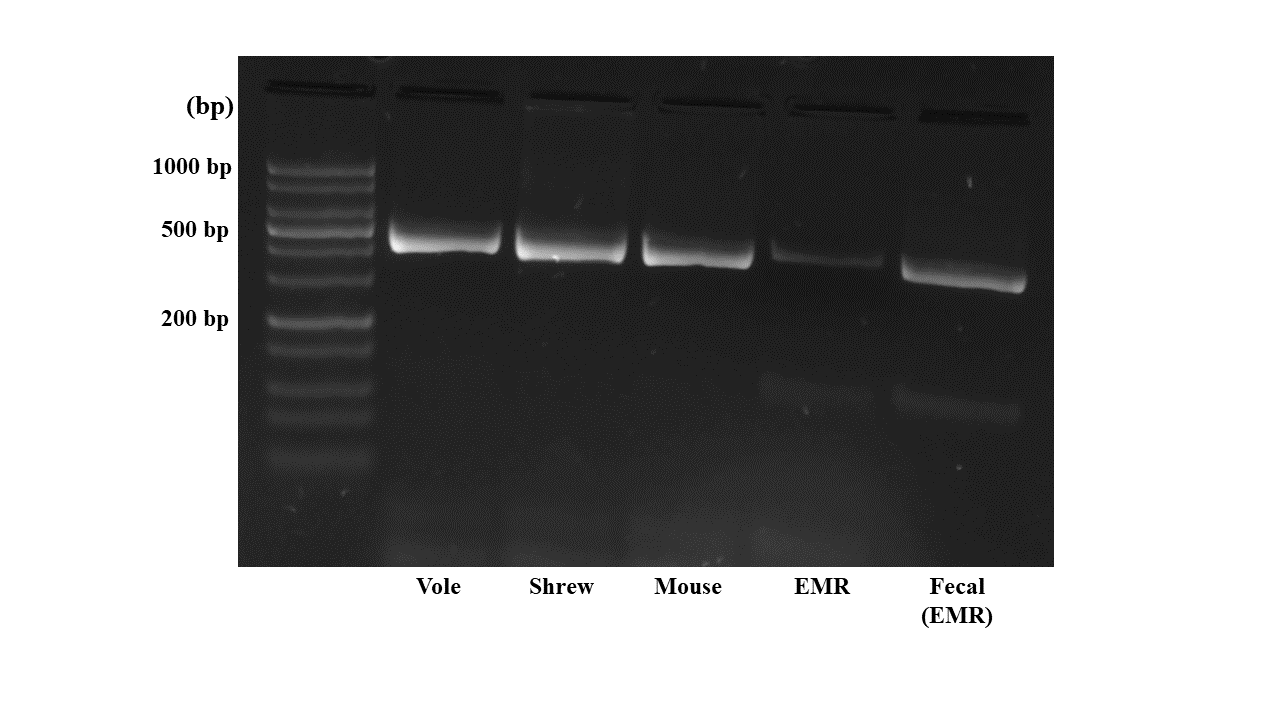
**

Figure S2. NMDS Jaccard matrix of diet items among young, juvenile, and adult age classes (P=0.076, stress= 0.04). We limited this analysis to prey items that had >1 occurrence across all samples (*Zapus hudsonius*, *Peromyscus leucopus*, *Napaeozapus insignis*, *Blarina brevicauda*, *Condylura cristata*, *Sorex cinereus*, *Microtus pennsylvanicus*).


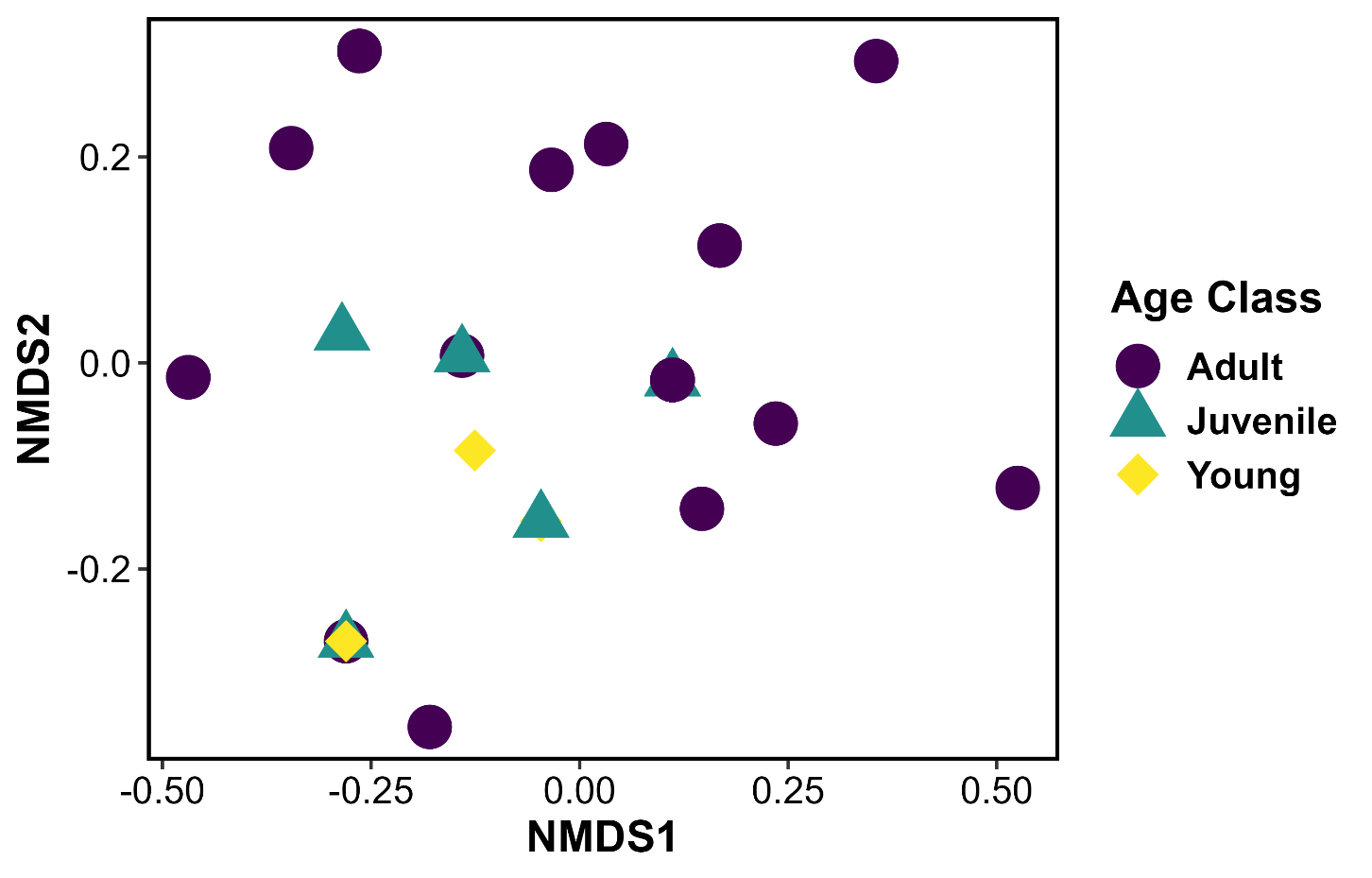


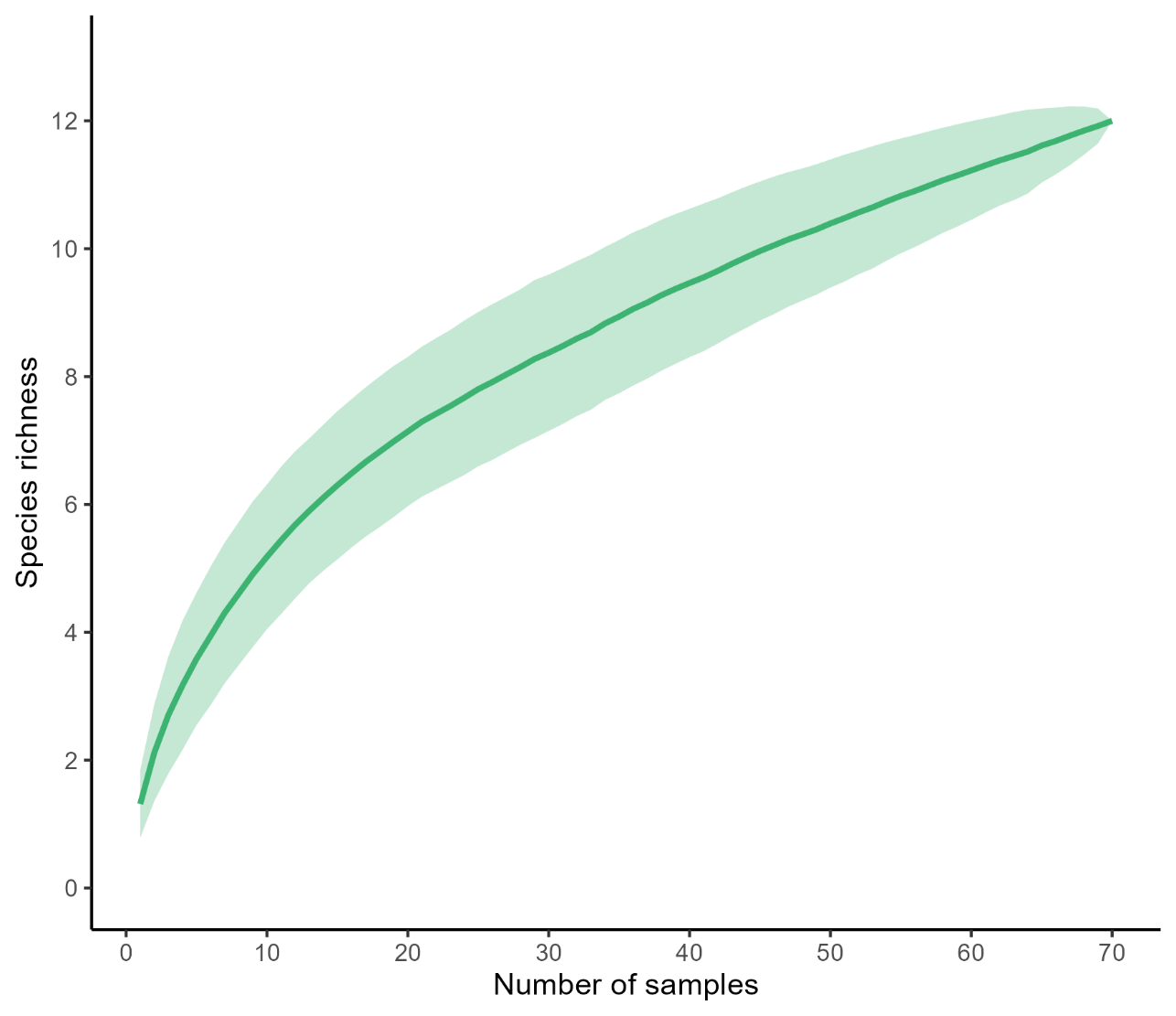
Figure S3. Species accumulation curve based on the number of fecal samples and presence/absence of prey taxa detected in the eastern massasauga dietary dataset. Shading represents standard deviation.


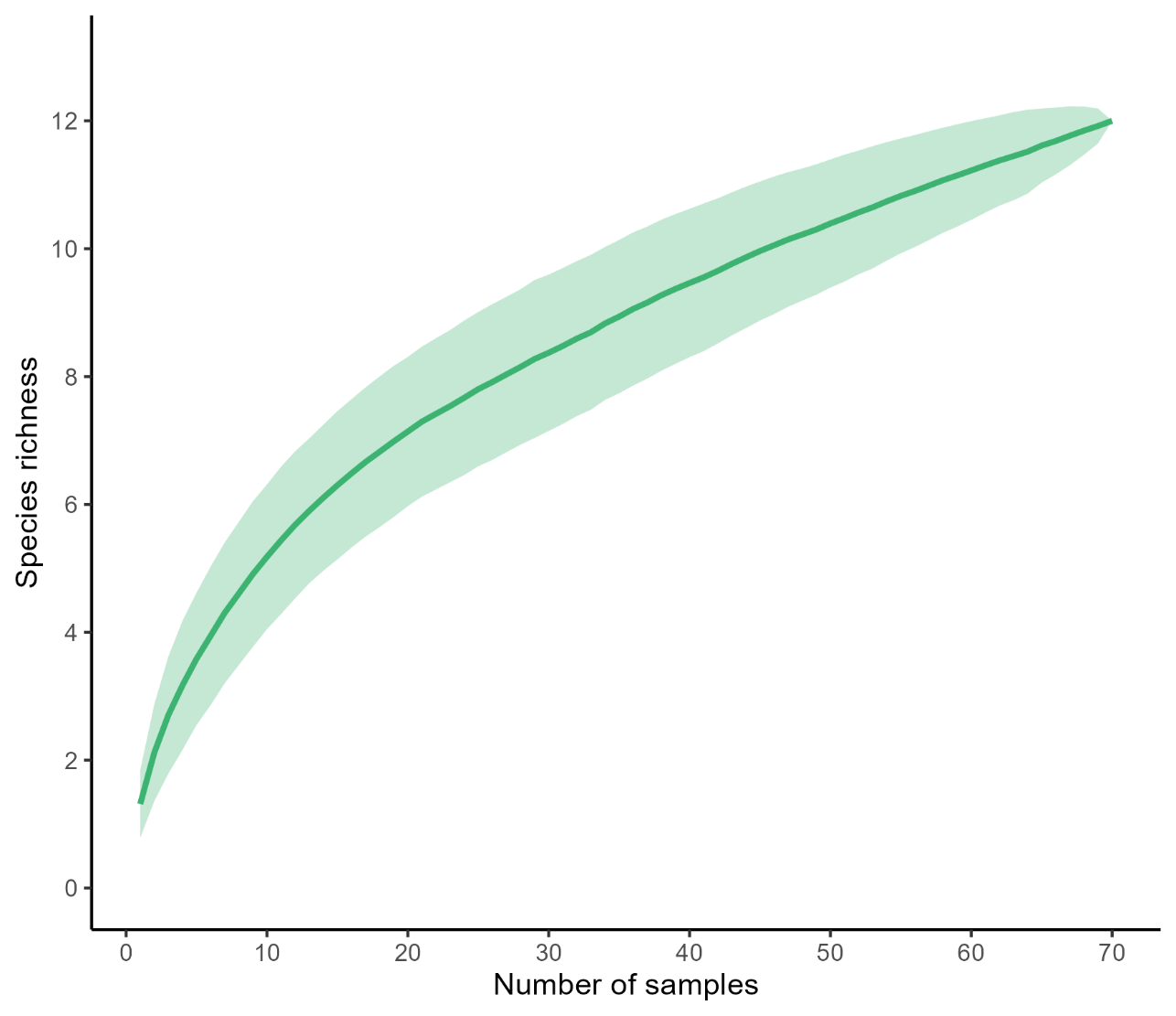


Table S1. List of potential prey species along with their accession number, from which the annealing inhibiting blocking oligonucleotide was designed. Sequences were aligned against the eastern massasauga in MEGA to locate sources of mismatches at the 3’ end.

| **Accession number** | **Species name** | **Common name** |
| --- | --- | --- |
| MG422537.1 | *Sorex cinereus* | Masked shrew |
| JF456798.1 | *Microtus pennsylvanicus* | Meadow vole |
| JF456964.1 | *Napaeozapus insignis* | Woodland jumping mouse |
| JF457177.1 | *Zapus hudsonius* | Meadow jumping mouse |
| JF435981.1 | *Blarina brevicauda* | Northern short-tailed shrew |
| JQ601063.1 | *Sylvilagus floridanus* | Eastern cottontail |
| JF457161.1 | *Tamias striatus* | Eastern chipmunk |
| JF457151.1 | *Tamiasciururs hudsonicus* | American red squirrel |
| JF457111.1 | *Sciurus niger* | Fox squirrel |
| JF457030.1 | *Peromyscus maniculatus* | Deer mouse |
| JF456922.1 | *Myodes gapperi* | Southern red-backed vole |
| JF456597.1 | *Glaucomys sabrinus* | Northern flying squirrel |
| GBMA1538-17 | *Lepus americanus* | Snowshoe hare |
| MN135612.1 | *Rana clamitans* | Green frog |
| MG422343.1 | *Pseudacris crucifer* | Spring peeper |
| EF525895.1 | *Rana septentrionalis* | Mink frog |
| EF5258861.1 | *Rana catesbeiana* | American bullfrog |
| EF525818.1 | *Hyla versicolor* | Gray treefrog |
| EF525740.1 | *Bufo americanus* | American toad |
| AAY666391.1 | *Agelaius phoeniceus* | Red-winged black bird |
| KU985793.1 | *Plestiodon fasciatus* | Five-lined skink |
| MH273655 | *Coluber constrictor* | Blue racer |
| MH274240 | *Lamropeltis triangulum* | Eastern milk snake |
| KU985887 | *Storeria dekayi* | Brown snake |
| KU986171 | *Clonophis kirtlandii* | Kirtland's snake |
| KU985725 | *Storeria occipitomaculata* | Red-bellied snake |
| KU985824 | *Opheodrys vernalis* | Smooth green snake |
| MH273770 | *Diadophis punctatus* | Ring-necked snake |
| KU986143 | *Thamnophis sauritus* | Northern ribbon snake |
| MH274704 | *Thamnophis sirtalis* | Eastern garter snake |
| MH274511 | *Nerodia sipedon* | Northern water snake |
| MH274129 | *Heteredon platirhinos* | Eastern hognose snake |

Table S2. Alignment of the eastern massasauga specific blocking primer (EMR_mlCOIintF_BLK) designed from the forward COI primer with potential snake prey. Dots indicate a shared nucleotide with the blocking primer, which may inadvertently block potential snake prey. Accession numbers represent unique sequence identifier from GenBank database.

| **Accession number** | **Species name** | **Sequences (5'-3')** | | | | | | | | | | | | | | | | | | | | | | | | | | | | |
| --- | --- | --- | --- | --- | --- | --- | --- | --- | --- | --- | --- | --- | --- | --- | --- | --- | --- | --- | --- | --- | --- | --- | --- | --- | --- | --- | --- | --- | --- | --- |
| EMR_mlCOIintF_BLK | | T | T | T | A | T | C | C | C | C | C | C | C | T | C | T | C | C | G | G | A | A | A | T | C | T | A | G | T | C |
| MH273655 | *Coluber constrictor* | **.** | **.** | **.** | **.** | C | **.** | **.** | **.** | **.** | **.** | A | **.** | **.** | A | **.** | **.** | T | **.** | **.** | **.** | **.** | **.** | **.** | T | **.** | **.** | **.** | **.** | **.** |
| MH274240 | *Lamropeltis triangulum* | **.** | C | **.** | **.** | **.** | **.** | **.** | A | **.** | **.** | **.** | **.** | **.** | G | **.** | **.** | T | **.** | **.** | **.** | **.** | **.** | **.** | **.** | **.** | **.** | **.** | **.** | A |
| KU985887 | *Storeria dekayi* | **.** | A | **.** | **.** | C | **.** | **.** | **.** | **.** | **.** | A | **.** | **.** | **.** | **.** | **.** | A | **.** | **.** | **.** | **.** | **.** | C | **.** | **.** | **.** | **.** | **.** | T |
| KU986171 | *Clonophis kirtlandii* | **.** | G | **.** | **.** | C | **.** | **.** | **.** | **.** | **.** | A | **.** | **.** | **.** | **.** | **.** | A | **.** | **.** | **.** | **.** | **.** | C | **.** | **.** | **.** | **.** | **.** | A |
| KU985725 | *Storeria occipitomaculata* | **.** | A | **.** | **.** | C | **.** | **.** | **.** | **.** | **.** | A | **.** | **.** | T | **.** | **.** | A | **.** | **.** | **.** | **.** | **.** | C | **.** | **.** | **.** | **.** | **.** | A |
| KU985824 | *Opheodrys vernalis* | **.** | **.** | **.** | **.** | **.** | **.** | **.** | A | **.** | **.** | T | T | **.** | G | **.** | **.** | A | **.** | **.** | **.** | **.** | **.** | C | **.** | **.** | **.** | **.** | **.** | A |
| MH273770 | *Diadophis punctatus* | **.** | C | **.** | **.** | **.** | **.** | **.** | **.** | **.** | **.** | T | T | **.** | A | **.** | **.** | **.** | **.** | **.** | **.** | **.** | **.** | C | T | **.** | **.** | **.** | **.** | A |
| KU986143 | *Thamnophis sauritus* | **.** | G | **.** | **.** | C | **.** | **.** | A | **.** | **.** | T | **.** | **.** | T | **.** | **.** | A | **.** | **.** | G | **.** | **.** | **.** | **.** | **.** | **.** | **.** | **.** | A |
| MH274704 | *Thamnophis sirtalis* | **.** | A | **.** | **.** | C | **.** | **.** | G | **.** | **.** | A | **.** | **.** | T | **.** | **.** | A | **.** | **.** | G | **.** | **.** | C | **.** | **.** | **.** | **.** | **.** | A |
| MH274511 | *Nerodia sipedon* | **.** | C | **.** | **.** | C | **.** | **.** | A | **.** | **.** | A | **.** | **.** | **.** | **.** | **.** | A | **.** | **.** | G | **.** | **.** | C | **.** | **.** | G | **.** | **.** | A |
| MH274129 | *Heteredon platirhinos* | **.** | G | **.** | **.** | C | **.** | **.** | T | **.** | **.** | T | **.** | **.** | A | **.** | **.** | A | **.** | **.** | G | **.** | **.** | C | **.** | **.** | **.** | **.** | **.** | T |
